# 

**Published:** 1868-02

**Authors:** 


### RECEIPTS FOR THE JOURNAL.

Drs. E. A. Flewellen, $4; J. H. Speir, $8; S. L. Richardson, $4 50; Wm. O'Daniel, $4 50; Geo. A. Hoke, $4 50; John T. Dixon, $4; B. Franklin, $4; E. A. Wall, $4; G. P.

Cosby, $4; H. S. McNary, $4; W. Morrow, $1. H. D. Hudson, $4; J. C. Turnipseed, $4; M. G. Williams, $4; J. T. Hester, $4; J. C. Harris, $1 50; N. T. Howard, $4; G. M. McDowell, $8; W. T. Crawford, $2; R. Searcy, $10; W. V. Aderhold, $8; W. Tally, $1 30; W. B. Wells, $8 36; B. F. Ree, $4; J. M. McLaughlin, $2 50; J. F. M. Davis, $8; G. T. Pursley, $5; W. B. Baker, $4; David Yarbrough, $5; N. B. Drewry, $4; John Dickinson, $4; J. O. Sanders, $4, A. H. Shi, $8; A. North, $3; J. M. Boring, $4; James Taylor, $10; Drs. Long, $4; E. D. Newton, $8; Hugh Harris, $6; J. C. Phelps, $4; J. B. Warren, $4; J. E. Dupree, $4; J. C. Avery, $4; G. W. Pinkston, $5; Thomas C. Gower, A. J. Gibbs, $4; Dr. Moore, $4; Dr. Carlton, $4; S. V. Childers, $8; G. W. Powell, $5; Rudicil & Calhoun, $3.

